# Position of Neocortical Neurons Transfected at Different Gestational Ages with shRNA Targeted against Candidate Dyslexia Susceptibility Genes

**DOI:** 10.1371/journal.pone.0065179

**Published:** 2013-05-28

**Authors:** William T. Adler, Maryann P. Platt, Alison J. Mehlhorn, Joshua L. Haight, Timothy A. Currier, Mikel A. Etchegaray, Albert M. Galaburda, Glenn D. Rosen

**Affiliations:** Department of Neurology, Beth Israel Deaconess Medical Center, Boston, Massachusetts, United States of America; University of Turin, Italy

## Abstract

Developmental dyslexia is a language learning disorder that affects approximately 4–10% of the population. A number of candidate dyslexia susceptibility genes have been identified, including *DCDC2* and *KIAA0319* on Chromosome (Chr) 6p22.2 and *DYX1C1* on Chr 15q21. Embryonic knockdown of the function of homologs of these genes in rat neocortical projection cell progenitors by *in utero* electroporation of plasmids encoding small hairpin RNA (shRNA) revealed that all three genes disrupted neuronal migration to the neocortex. Specifically, this disruption would result in heterotopia formation (*Dyx1c1* and *Kiaa0319*) and/or overmigration past their expected laminar location (*Dyx1c1* and *Dcdc2*). In these experiments, neurons normally destined for the upper neocortical laminæ were transfected on embryonic day (E) 15.5, and we designed experiments to test whether these migration phenotypes were the result of targeting a specific type of projection neuron. We transfected litters with *Dcdc2* shRNA, *Dyx1c1* shRNA, *Kiaa0319* shRNA, or fluorescent protein (as a control) at each of three gestational ages (E14.5, E15.5, or E16.5). Pups were allowed to come to term, and their brains were examined at 3 weeks of age for the position of transfected cells. We found that age of transfection did not affect the percentage of unmigrated neurons—transfection with *Kiaa0319* shRNA resulted in heterotopia formation at all three ages. Overmigration of neurons transfected with *Dcdc2* shRNA, while present following transfections at the later ages, did not occur following E14.5 transfections. These results are considered in light of the known functions of each of these candidate dyslexia susceptibility genes.

## Introduction

Developmental dyslexia, a language learning disorder, affects approximately 4–10% of the global population and has a strong genetic component. In the past decade, a number of candidate dyslexia susceptibility genes (CDSGs) have been identified, three of which have garnered significant support in diverse populations, including *DCDC2* and *KIAA0319* on Chromosome (Chr) 6p22.2 [1]–[16] and *DYX1C1* on Chr 15q21 [17]–[22]. Delineating the specific functions of these genes has been a target of intense investigation in recent years. For example, these three CDSGs have been shown to be involved in neocortical neuronal migration [2], [23]–[32]. This role in neuronal migration is particularly intriguing given the association of neuronal migration disorders such as molecular layer ectopias and periventricular nodular heterotopias with developmental dyslexia in humans [33]–[37].

Previous work demonstrated that disruption of CDSG homolog function at embryonic day (E) 15.5 potentially yields two phenotypes in the postnatal rat. Specifically, *in utero* electroporation of plasmids encoding shRNA targeted against *Dyx1c1* at E15.5 resulted in one cohort of neurons that remained in the white matter (heterotopia) and another that migrated past their expected laminar location (overmigration) [25], [26]. In contrast, only heterotopias were seen following identical transfection with plasmids encoding *Kiaa0319* shRNA [30], whereas knockdown of *Dcdc2* by the same method resulted in the overmigration phenotype only [24]. In these experiments, neurons normally destined for the upper neocortical laminæ were transfected, which raises a number of questions: Are the migration phenotypes the result of targeting a specific type of projection neuron? What would be the effect of targeting neurons destined for laminæ both below (layers 5–6) and above (layer 2)? In this experiment, we transfected litters with *Dcdc2* shRNA, *Dyx1c1* shRNA, *Kiaa0319* shRNA, or fluorescent protein (as a control) at one of three gestational ages (E14.5, E15.5, or E16.5). Pups were allowed to come to term, and their brains were examined at 3 weeks of age for the position of transfected cells. We found that age of transfection did not affect the percentage of unmigrated neurons, but did influence the overmigration phenotype.

## Experimental Procedures

### *In utero* electroporation

All procedures were approved by the Institutional Animal Care and Use Committee at Beth Israel Deaconess Medical Center. Time-mated pregnant Wistar rats (Charles River, Wilmington, MA, USA) were assigned to one of three experimental shRNA conditions: *Dcdc2* shRNA, *Dyx1C1* shRNA, or *Kiaa0319* shRNA. Each mother was also assigned to one of three transfection age conditions: E14.5, E15.5, or E16.5. Within each litter, about twice as many pups received an experimental treatment (shRNA + mRFP) as received a control treatment (eGFP). *In utero* electroporations were performed as previously described [26].

#### Plasmids

For the experimental shRNA condition, plasmids encoding shRNA (pU6DyxHPB, pU6shRNA-Kiaa0319, or pU6shRNA-Dcdc2A) and plasmids encoding mRFP (pCAGGS-mRFP) were co-transfected. Previous research indicates that co-transfection is highly efficient [26]. Littermates in the control condition were transfected with a plasmid encoding eGFP (pCAGGS-eGFP). The concentrations of eGFP and mRFP plasmids were 0.75 µg/ µL, and the shRNA construct concentrations used were 1.5 µg/ µL.

### Histology

At postnatal day (P) 21, transfected rats were deeply anesthetized (Ketamine/Xylazine 10∶1, 100 mg/mL), sacrificed, and fixed by transcardial perfusion with 0.9% saline followed by 4% paraformaldehyde. Brains were extracted, post-fixed for 24 h, and cryoprotected, first in 10% and then in 30% sucrose phosphate buffer. The tissue was frozen and sectioned at 40 µm on a sliding microtome. Sections were stored in series of every tenth section and preserved in 0.4% sodium azide/phosphate buffer. One series was then mounted and visualized under fluorescence for the presence of eGFP or mRFP. After screening, that series was stained for Nissl bodies using Thionin.

#### Immunohistochemistry

Immunoperoxidase activity was detected using 3,3-diaminobenzidine (DAB,Vector Labs) according to ABC protocols. One series adjacent to the Nissl-stained sections was used for the immunohistochemical detection of eGFP (AB3080, Millipore Corp., Billerica, MA, USA, 1∶1800) or mRFP (18-732-292379, Genway Biotech, San Diego, CA, USA, 1∶5000). Primary antibodies were labeled with biotinylated secondary antibodies (Vector Labs, Burlingame, CA, all 1∶200). Immunohistochemically processed tissue was mounted, counterstained with Methyl Green/Alcian Blue, and coverslipped with Permount mounting medium (Fisher Scientific, Waltham, MA, USA). Selected tissue from transfection ages E14.5 and E16.5 was reacted for immunofluorescence of laminar markers CUX1 and FOXP2 (SC-13024, 1∶1000; SC-21069, 1∶50, respectively, Santa Cruz Biotechnology, Santa Cruz, CA, USA). It should be noted that although CTIP2 reliably labels neurons in layer 5 in the mouse, it is, in our hands, expressed most strongly in layer 6, and inconsistently in layers 2 and 5 (Figure S1). Primary antibodies were detected with fluorescent secondary antibodies AlexaFluor 488 or AlexaFluor 594 (Invitrogen, Carlsbad, CA, USA, all 1∶200). Tissue processed for immunofluorescence were mounted, coverslipped with VECTASHIELD HardSet Mounting Medium (Vector Labs, Burlingame, CA, USA), and imaged.

### Analysis

#### Assessment of pathology

All brains were analyzed with the experimenter blind to subject condition. Nissl-stained sections were surveyed for the presence of neocortical malformations.

#### Quantitative analysis of neuronal position

For each brain, four sections were chosen from the eGFP/mRFP immunostained series. The positions of immunohistochemically stained neurons were charted using Neurolucida (MBF Biosciences, Williston, VT, USA). The charted sections were then analyzed in a custom MATLAB (Mathworks, Natick, MA, USA) program that determined the location of each marked neuron as a percentage of cortical depth, with 0% corresponding to the white matter/neocortex boundary and 100% representing the pial surface [30]. The region analyzed was limited to the area of cortex that was transfected, mainly the primary somatosensory (S1) cortex. We determined the average percentage of neurons that did not migrate into the neocortex (heterotopic neurons), as well as the mean distance traveled into the neocortex for each group. Results were analyzed by ANOVA with Fisher LSD post-hoc tests.

### Image processing

Fluorescent images were obtained on a Zeiss LSM 5000 confocal microscope (Carl Zeiss, Inc., Thornwood, NY). Photomicrographs were adjusted for exposure and sharpened (unsharp mask filter) with Adobe Photoshop (Adobe Inc., San Jose, CA). Brightfield images were obtained on a Nikon E800 (Nikon Corporation, Melville, NY). All figures were composited in Adobe Illustrator (Adobe Inc.).

## Results

### Qualitative analysis

**Figure 1:**
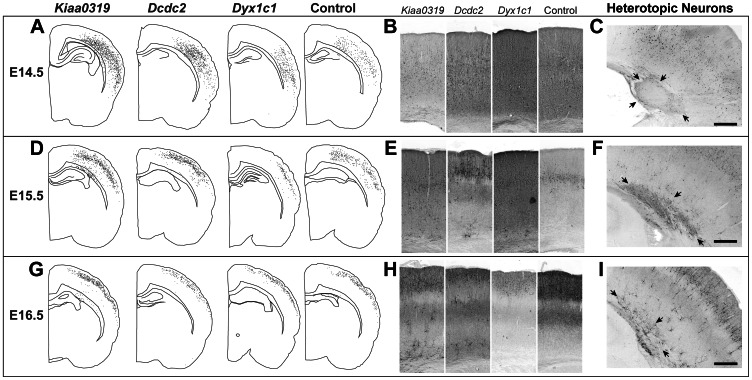
Hemispheric tracings (A,D,G), neocortical photomicrographs (B,E,H), and photomicrographs of white matter heterotopias (C,F,I) of rats embryonically transfected with either *Kiaa0319* shRNA, *Dcdc2* shRNA, *Dyx1c1* shRNA, or fluorescent protein (Control) at either E14.5 (A,B,C), E15.5 (D,E,F), or E16.5 (G,H,I). Bar for C,F,I = 500 µm.

The position of transfected neurons varied considerably based on the day of transfection, and to a lesser extent on the construct being transfected. Labeled neurons from rats embryonically transfected with *Kiaa0319*, *Dcdc2*, or *Dyx1c1* shRNA at E14.5 were found predominantly in layers 4–6 of the cortex (Fig 1A,B). The disposition of transfected neurons migrating to the cerebral cortex did not appear to differ from the controls (n = 10). White matter heterotopias were seen in 7/8 of the brains transfected with *Kiaa0319* shRNA. In contrast, none of the 5 rats embryonically transfected with *Dcdc2* shRNA and 1/7 rats transfected with *Dyx1c1* shRNA had heterotopias. There were no heterotopias in the control condition.

**Figure 2:**
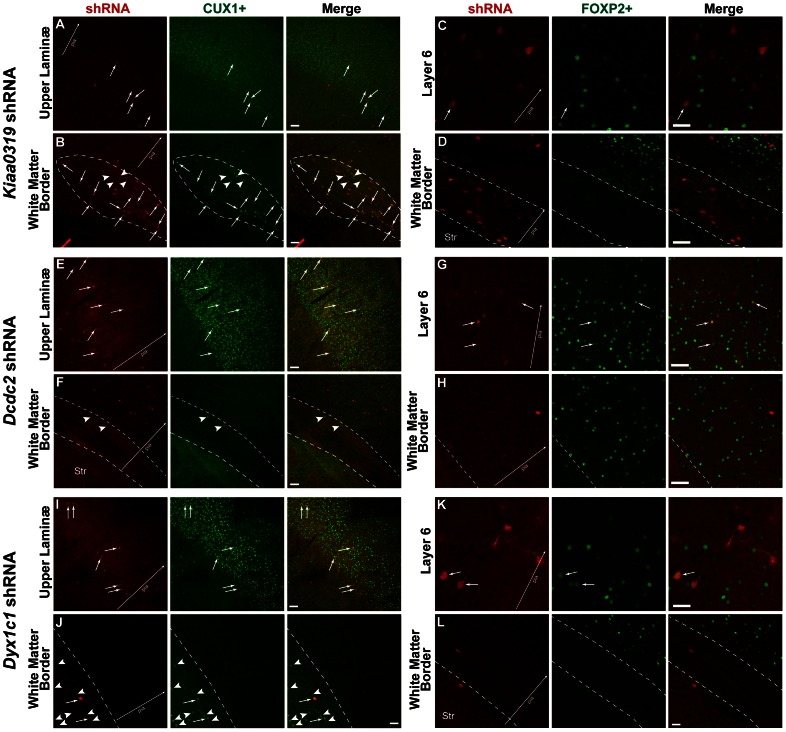
Confocal microscopy of laminar markers CUX1 and FOXP2 following transfection of candidate dyslexia susceptibility genes at E14.5. Small arrows indicate transfected cells co-labeled with CUX1 or FOXP2. Arrowheads indicate CUX1+ neurons that are not transfected. Large arrow for orientation with pial surface. Bar = 200 µm in all panels. **A**. Photomicrograph of upper lamina neurons from a rat transfected with *Kiaa0319* shRNA. **B**. Photomicrograph of white matter heterotopia (dotted line) from a rat transfected with *Kiaa0319* shRNA. **C**. Photomicrograph of layer 6 neurons from a rat transfected with *Kiaa0319* shRNA. **D**. Photomicrograph of white matter heterotopia (dotted line) from a rat transfected with *Kiaa0319* shRNA. **E**. Photomicrograph of upper lamina neurons from a rat transfected with *Dcdc2* shRNA. **F**. Photomicrograph of white matter border (dotted line outlines corpus callosum) from a rat transfected with *Dcdc2* shRNA. Str = Striatum. **G**. Photomicrograph of layer 6 neurons from a rat transfected with *Dcdc2* shRNA. **H**. Photomicrograph of white matter border (dotted line) from a rat transfected with *Dcdc2* shRNA. **I**. Photomicrograph of upper lamina neurons from a rat transfected with *Dyx1c1* shRNA. **J**. Photomicrograph of white matter heterotopia (dotted line is white matter border) from a rat transfected with *Dyx1c1* shRNA. **K**. Photomicrograph of layer 6 neurons from a rat transfected with *Dyx1c1* shRNA. **L**. Photomicrograph of white matter border (dotted line) from a rat transfected with *Dyx1c1* shRNA. Str = Striatum

We examined brains from each of the E14.5 transfection conditions for the presence of CUX1 (layers 2–4) and FOXP2 (layer 6) immunopositive neurons in the somatosensory cortex (Fig 2). Of the relatively few transfected neurons that migrated to layer 4, most were co-labeled with CUX1 (Fig 2A,E,I), indicating that these neurons are expressing markers appropriate for their lamina. We found substantial numbers of CUX1+ neurons in the white matter heterotopias, only a fraction of which were transfected with shRNA (Fig 2B,J). The large number of untransfected CUX1+ neurons in these heterotopias supports the notion that there are non-cell autonomous effects of *in utero* electroporation of shRNA as we have described previously for E15.5 transfected neurons [25], [30]. FOXP2+ neurons were found, as expected, in layer 6 of the neocortex in all transfection conditions. A few of these neurons were transfected (Fig 2C,G,K). In contrast to CUX1+ neurons, there was no evidence of FOXP2+ neurons in the white matter heterotopia (Fig 2D,H,L). Taken together, these results suggest that white matter heterotopias that occur following E14.5 transfection with shRNAs targeted against CDSGs are composed of late generated neurons destined for the upper laminæ.

*In utero* electroporation of plasmids containing shRNA targeted against *Kiaa0319*, *Dcdc2*, or *Dyx1c1* at E15.5 mostly replicated previous reports [23]–[26], [30]. Specifically, embryonically transfected neurons (both shRNA and control (n = 14)) that migrated into the neocortex were found predominantly in layers 2–4 (Fig 1C,D) in all conditions. White matter heterotopias were found in 6/10 of brains transfected with *Kiaa0319* shRNA, but in none of those embryonically transfected with *Dcdc2* shRNA (n = 5). Although rats *in utero* electroporated with shRNA targeted against *Dyx1c1* had scattered collections of unmigrated neurons at the border of the white matter and neocortex, only 1/5 rats had frank subcortical heterotopias, which is less than the expected incidence from previous studies [23], [25], [26]. The dispositions of CUX1+ and FOXP2+ neurons were identical to those previously published. Specifically, there were large numbers of transfected neurons co-labeled with CUX1 in layers 2–4 of the neocortex, whereas there were no transfected neurons co-labeled with FOXP2. In those rats with white matter heterotopias, there were large numbers of CUX1+ neurons, only a subset of which were transfected, which is consistent with there being non-cell autonomous effects of embryonic knockdown of *Kiaa0319* or *Dyx1c1* [25], [26], [30]. There were no FOXP2+ neurons in the heterotopia, again suggesting that heterotopic neurons are generated in the later stages of gestation.

Late transfections (E16.5) resulted in labeled neurons (both shRNA and control (n = 10)) being found almost exclusively in the upper portion of neocortical layer 2–3 (Fig 1E,F). White matter heterotopias were seen in 7/8 of the brains embryonically transfected with *Kiaa0319* shRNA, compared with 2/10 following *in utero* electroporation of *Dcdc2* and 2/6 *Dyx1c1* shRNA.

**Figure 3:**
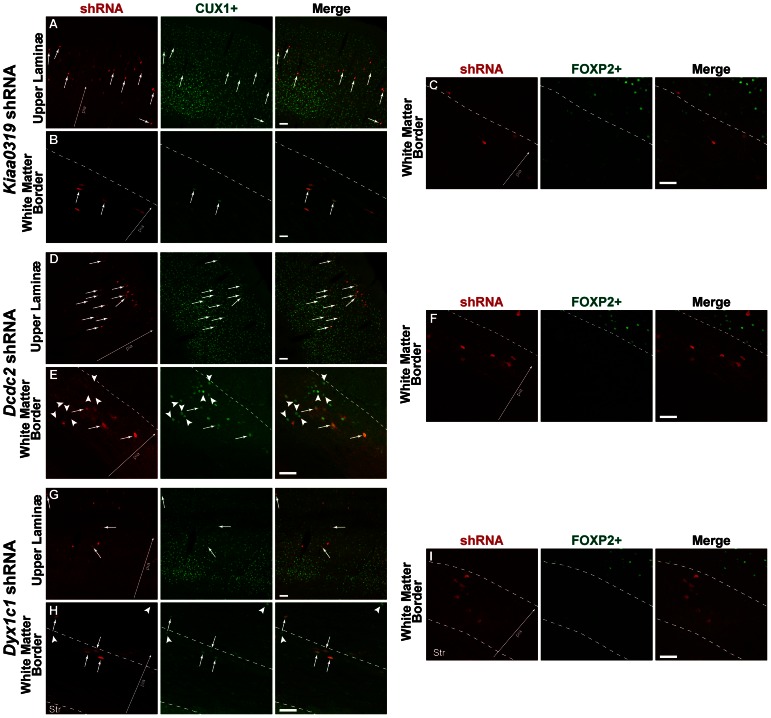
Confocal microscopy of laminar markers CUX1 and FOXP2 following transfection of candidate dyslexia susceptibility genes at E16.5. Small arrows indicate transfected cells co-labeled with CUX1 or FOXP2. Arrowheads indicate CUX1+ neurons that are not transfected. Large arrow for orientation with pial surface. Bar = 200 µm in all panels. **A**. Photomicrograph of upper lamina neurons from a rat transfected with *Kiaa0319* shRNA. **B**. Photomicrograph of white matter border (dotted line) from a rat transfected with *Kiaa0319* shRNA. **C**. Photomicrograph of white matter border (dotted line) from a rat transfected with *Kiaa0319* shRNA. **D**. Photomicrograph of upper lamina neurons from a rat transfected with *Dcdc2* shRNA. **E**. Photomicrograph of white matter border (dotted line) from a rat transfected with *Dcdc2* shRNA. **F**. Photomicrograph of white matter border (dotted line) from a rat transfected with *Dcdc2* shRNA. **G**. Photomicrograph of upper lamina neurons from a rat transfected with *Dyx1c1* shRNA. **H**. Photomicrograph of white matter heterotopia (dotted line) from a rat transfected with *Dyx1c1* shRNA. Str = Striatum. **I**. Photomicrograph of white matter heterotopia (dotted line) from a rat transfected with *Dyx1c1* shRNA. Str = Striatum

Of the E16.5 transfected neurons that migrated to layer 2–3, many (but not all) were co-labeled with CUX1, which was true for all transfection conditions. (Fig 3A,D,G). There were CUX1+ neurons in the white matter heterotopias, and some of these were embryonically transfected with shRNA (Fig 3B,E,H). FOXP2+ neurons were found, as expected, in layer 6 of the neocortex in all transfection conditions, and none of these were transfected. There were no FOXP2+ neurons in the white matter heterotopia (Fig 3C,F,I). Taken together, these results suggest that white matter heterotopias that occur following E16.5 transfection with shRNAs targeted against CDSGs, for both transfected and non-cell autonomous effects, are composed of late generated neurons destined for the upper laminæ.

### Quantitative analysis

**Figure 4:**
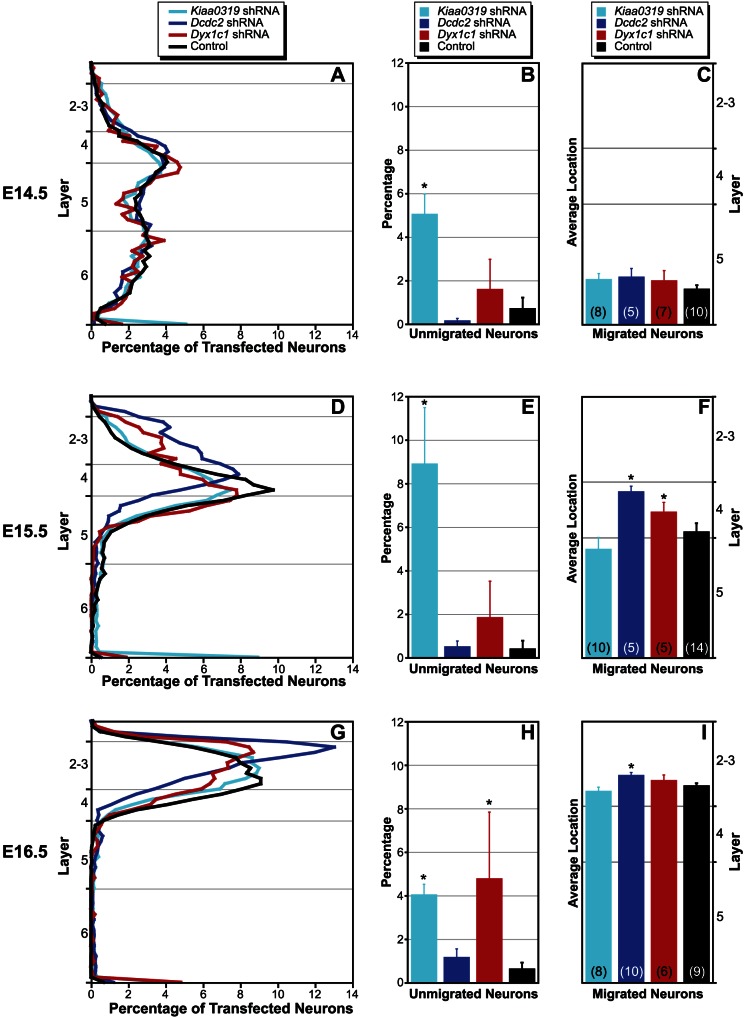
Quantitative analysis of transfected neuron position. **A**. Line chart showing mean laminar position of neurons transfected at E14.5. **B**. Histogram illustrating the mean percentage (+SEM) of transfected neurons that are unmigrated following E14.5 transfection. *Differs from control, P<.05. **C**. Histogram of mean (+SEM) average position of transfected neurons that migrated into the neocortex following E14.5 transfection. Horizontal lines indicate laminar boundaries. **D**. Line chart showing mean laminar position of neurons transfected at E15.5. **E**. Histogram illustrating the mean percentage (+SEM) of transfected neurons that are unmigrated following E15.5 transfection. *Differs from control, P<.05. **F**. Histogram of mean (+SEM) average position of transfected neurons that migrated into the neocortex following E15.5 transfection. Horizontal lines indicate laminar boundaries. *Differs from control, P<.05. **G**. Line chart showing mean laminar position of neurons transfected at E16.5. **H**. Histogram illustrating the mean percentage (+SEM) of transfected neurons that are unmigrated following E16.5 transfection. *Differs from control, P<.05. **I**. Histogram of mean (+SEM) average position of transfected neurons that migrated into the neocortex following E16.5 transfection. Horizontal lines indicate laminar boundaries.*Differs from control, P<.05.

We used a customized MATLAB program to quantify the position of each transfected neuron in 4 sections taken from each case. The position of each neuron is given as a percentage of cortical depth, with 0% being the border with the white matter and 100% being the pial surface. In the E14.5 transfection group, the plot reflects the qualitative assessment, with the majority of transfected neurons in each condition found in layers 4–6 of the neocortex (Fig 4A). We computed the percentage of total transfected neurons that were unmigrated, and ran an ANOVA with Condition (4 levels) as the independent variable (Fig 4B). We found a significant main effect of Condition (*F*_2,26_ = 6.6, *P*<.01) on the average number of unmigrated neurons, and post-hoc analysis revealed that there were more unmigrated neurons in the *Kiaa0319* group as compared to the control (Fisher LSD *t* = 4.3, *P*<.001). We next computed the average distance of the neurons that migrated into the neocortex (Fig 4C). Transfected neurons were found predominantly in layers 4–6, and a one-way ANOVA revealed no significant difference in position among the four groups (*F*<1, NS).

Neurons transfected at E15.5 were distributed predominantly in the upper laminae in a manner similar to that seen in previous reports (Fig 4D), and analyses are therefore one-tailed. There was a significant difference in the percentage of unmigrated neurons (*F*_3,30_ = 7.2, *P*<.001), with there being a significantly greater percentage of unmigrated neurons in the *Kiaa0319* group as compared to controls (Fisher LSD *t = *6.1, *P*<.001; Fig 4E). There was also a significant difference in the average location of neurons that migrated to the neocortex (*F*_3,30_ = 4.9, *P*<.01). In this case, both the *Dcdc2* and *Dyx1c1* groups migrated past the controls (Fisher LSD *t* = 4.3, *P*<.01, *t* = 1.6, *P*<.06, respectively; Fig 4F).

The migration plot for the E16.5 transfection groups reveals that nearly all neurons that migrated into the cortex were found in layer 2–3 (Fig 4G). There was a significant effect of experimental group in the percentage of unmigrated neurons (*F*_3,29_ = 3.1, *P*<.05), with both *Kiaa0319* and *Dyx1c1* having significantly higher percentage of unmigrated neurons than controls (Fisher LSD *t* = 2.2, *P*<.05, *t* = 2.4, *P*<.05, respectively; Fig 4H). Fig 4I confirms that the average position of migrated neurons in the neocortex is in layer 2–3 for all groups. There was a significant effect of group (*F*_3,29_ = 5.2, *P*<.01), with *Dcdc2* showing significant overmigration (Fisher LSD *t* = 2.5, *P*<.05).

## Discussion

Previous research demonstrated that embryonic knockdown of candidate dyslexia susceptibility gene (CDSG) homologs at E15.5 results in disruption of neuronal migration, including white matter heterotopias and/or migration of transfected neurons past their expected laminar location in the neocortex. Here we report the effects of *in utero* electroporation of plasmids encoding shRNA targeted against the CDSG homologs *Kiaa0319*, *Dcdc2*, and *Dyx1c1* at each of three embryonic ages (E14.5, E15.5, and E16.5) on the eventual disposition of transfected neurons in the neocortex. We successfully replicated the results of the previous reports using E15.5 transfection: *Kiaa0319* knockdown typically resulted in a group of unmigrated neurons in the white matter, and the neurons that did migrate into the neocortex were positioned identically to control transfected neurons [30]. Neurons embryonically transfected with *Dcdc2* shRNA, on the other hand, migrated past their expected laminar location, while few were unmigrated [24]. Transfection with *Dyx1c1* shRNA resulted in one case of white matter heterotopia, and moderate numbers of small groups or isolated unmigrated neurons that did not coalesce into a heterotopia. In addition, *Dyx1c1* shRNA transfected neurons migrated past their expected location [23], [25], [26]. In all cases, the majority of transfected neurons were CUX1 positive and FOXP2 negative, indicating they were mostly supragranular neurons. In cases where there were white matter heterotopias (*Kiaa0319* and *Dyx1c1* shRNA), there were no FOXP2+ neurons, and only a subset of the CUX1+ neurons in the heterotopia were transfected, with the rest showing non-cell autonomous migration defects.

Transfection with fluorescent protein alone at E14.5 generally labeled neurons in all layers of the neocortex, with the heaviest concentration in layers 5 and 6. *In utero* electroporation with shRNA targeted against *Kiaa0319* resulted in heterotopic collections of neurons in the white matter. As with identical transfections that took place at E15.5, a subset of neurons in the heterotopias were transfected and were CUX1+, another subset were untransfected and CUX1+, and a further cohort were transfected and CUX1- [30]. In contrast, there were no large collections of unmigrated neurons following embryonic transfections with *Dcdc2* shRNA. Embryonic knockdown of *Dyx1c1* function at E14.5 resulted in white matter heterotopias in one rat, although there were clusters of transfected neurons at the white matter border in other animals. There was no evidence of an overmigration phenotype for any of the CDSGs following this early transfection—shRNA transfected neurons migrated to positions indistinguishable from those transfected with only fluorescent protein. In all CDSG conditions, subsets of neurons were co-labeled with CUX1 or FOXP2, again supporting the notion that transfection at this age labels neurons from the complete range of cortical laminæ.

Late (E16.5) transfections with fluorescent proteins labeled neurons exclusively in the upper cortical laminæ, with virtually no transfected neurons in layers 5 or 6. White matter heterotopias were seen in both the *Kiaa0319* and *Dyx1c1* conditions, and small heterotopias in 20% of the rats in the *Dcdc2* condition. There was a significant overmigration phenotype for the *Dcdc2* condition only, although there was some suggestion of supralaminar placement of *Dyx1c1* shRNA transfected neurons (Fig 4G) at this age. As with the E14.5 and E15.5 group, there were CUX1+ neurons in the heterotopias—only a subset of which were transfected—which again suggests non-cell autonomous effects of embryonic shRNA transfection on neuronal migration. There was no co-labeling of FOXP2+ and transfected neurons in any condition.

Taken together, these results suggest that disruption of neuronal migration following embryonic transfection with CDSG shRNA is partially dependent on the targeted neuronal population. In the case of white matter heterotopias, there appears to be little or no difference in incidence when age of transfection is taken into account. In the *Kiaa0319* shRNA transfected group, white matter heterotopias were seen at all transfection ages, with relatively high incidence regardless of age (∼60–90%). In contrast, there were only 2 (out of 20) cases of white matter heterotopias in all the *Dcdc2* shRNA conditions, and there was never any statistical difference in the percentage of unmigrated neurons between the controls and this group for any of the three transfection ages. Although the incidence of white matter heterotopias following *Dyx1c1* transfection was relatively low when compared to previous results [23], [25]–[28], the cases that did occur were rather large at all transfection ages and morphologically similar to previously reported findings.

In contrast, the overmigration phenotype is dependent on the age of transfection. Previous research had demonstrated that neurons embryonically transfected at E15.5 with either *Dcdc2* shRNA or *Dyx1c1* shRNA tended to migrate past their expected laminar targets [24], [26], whereas those transfected with *Kiaa0319* shRNA did not [30],results that were replicated in the current study. We did not, however, find evidence of an overmigration effect for any of the CDSG groups following transfection at E14.5. Interestingly, we did see an overmigration effect following E16.5 transfection, despite the fact that the position of control transfected neurons would seemingly leave little space for overmigration.

The process of neuronal migration in the developing neocortex is a complicated series of interactions, involving many different genes and precise timing of events. Progenitor cells located in the VZ give rise to the projection cells of the neocortex as well as providing the scaffolding (radial glial fibers) by which these newly generated neurons migrate into the cortical plate. These neurons must orient properly, attach to the radial glial fibers, and then migrate into the cortical plate in an inside-out manner, and take their proper laminar position before beginning to differentiate. Disruptions of any of the genes modulating this process, however, can result in a variety of neuronal migration disorders [38].

The different functions of these CDSGs on neuronal migration may provide insight as to their roles in the different phenotypes observed. KIAA0319 encodes an integral membrane protein with a large extracellular domain, a single transmembrane domain, and a small intracellular C-terminus. There are several splice variants of this gene, one of which is secreted [11], [31]. Because of its 5 polycystic kidney domains, it has been suggested that this gene plays a role in cell adhesion, and is therefore consistent with its being involved in neural cell adhesion [39]. DCDC2 is one of an eleven-member group of proteins distinguished by the presence of doublecortin domains [40]–[42]. The doublecortin domain is critical for binding to and stabilizing microtubules [42], and two members of this family (DCX and DCLK) have been shown to interactively affect axon outgrowth and neocortical neuronal migration [43], [44]. Overexpression of the N- terminal p23 domain of DYX1C1 protein can interact with Hsp70, Hsp90 and CHIP, all of which point toward its role in degradation of unfolded proteins [45], [46]. Recently, DYX1C1 has been shown to regulate estrogen receptors, which may have an impact on estrogen signaling during development [22], [47], [48]. Interestingly, all three CDSGs have recently been associated with cilia function [49].

KIAA0319 is a transmembrane protein that is purported to be a novel neural cell adhesion molecule. In comparison, DCDC2 is not an adhesion molecule, but is cytoplasmic and modulates microtubule function during development in a manner similar to other genes of the doublecortin family associated with neuronal migration disorders [38]. We have found that transfection with *Kiaa0319* shRNA at all three embryonic ages results in white matter heterotopias, whereas *Dcdc2* shRNA transfection rarely does. Of those *Kiaa0319* shRNA-transfected neurons that do migrate into the cerebral cortex, they do so in a manner indistinguishable from neurons transfected with fluorescent protein alone, whereas neurons that had *Dcdc2* function knocked down at E15.5 and 16.5 were positioned beyond their expected laminar location. It is therefore tempting to speculate that disruption of the purported adhesion molecule Kiaa0319 interferes with the initial attachment of some of the newly generated neurons to radial glia. In this case, those neurons would remain in the white matter, whereas the subpopulation that did attach migrated successfully into the neocortex and achieved their expected laminar position.

In contrast, almost all *Dcdc2*-transfected neurons migrated into the neocortex, and those neurons transfected at E15.5 and E16.5 tended to position themselves beyond their expected laminar position. The mechanisms that underlie this overmigration phenotype are not yet known, but any explanation must take into account a lack of this phenotype in E14.5 transfected neurons. One possible explanation is that the earlier transfected neurons that are destined for layers 5–6, which comprise over 60% of the cortical depth, would be more difficult to demonstrate to be out of position, since they are normally located in a broad swath of cortex. Later migrating neurons (layers 2–4), which end up more restricted in upper laminæ, would be more easily identified as being in an abnormal position. This is consistent with our findings.

Overmigration could be the result of cell-autonomous or non-cell autonomous effects of the transfection. We have previously demonstrated (and have replicated here) that there are non-cell autonomous effects of *in utero* electroporation of CDSG shRNA with respect to neurons in the heterotopia and we have previously discussed possible mechanisms in detail [23], [24], [26], [30]. One bit of evidence indicating non-cell autonomous effects is the presence of non-transfected GABAergic neurons in the heterotopia [25]. We did not find, however, any evidence of misplacement of GABAergic neurons in the cerebral cortex following embryonic transfection [25], suggesting that, at least in the case of these non-transfected neurons, there is no demonstrable evidence of non-cell autonomous effects on neuronal migration once they migrate into the cortex.

One potential mechanism of overmigration is that transfection delays the departure of transfected neurons from the ventricular zone, causing them to migrate with a cohort of later-generated neurons. Although the current study does not directly address this issue, the evidence is consistent with this hypothesis. Specifically, previous research demonstrated that there were large numbers of unmigrated *Dcdc2* shRNA-transfected neurons when examined 4 days post-transfection, with only a handful of neurons having migrated into the cortical plate [2], [9], [23]. This is in contrast with the controls, where most transfected neurons have migrated into the cortical plate by this time. Three weeks later, by contrast, there are relatively few unmigrated *Dcdc2* shRNA transfected neurons, but there are substantial numbers in the neocortex, and they have migrated past their expected laminar location. We therefore speculate that the embryonic disruption of Dcdc2 interferes with the normal activity of the migrating neurons, delaying their initial migration from the ventricular zone, causing them to migrate with later generated neurons and therefore taking positions beyond their expected location. Ongoing experiments documenting the developmental time course of neuronal migration following embryonic disruption of CDSGs will directly address these hypotheses.

## Supporting Information

Figure S1 **Laminar markers FOXP2 (A) and CTIP2 (B) in the somatosensory cortex.** FOXP2+ and CTIP2+ neurons are found predominantly in layer 6, although CTIP2 inconsistently labels layer 5 and layer 2 neurons as well. Bar = 500 µm.
(TIF)
