# Supplementary figures and images for: Position of Neocortical Neurons Transfected at Different Gestational Ages with shRNA Targeted against Candidate Dyslexia Susceptibility Genes
Source: PLoS One. 2013 May 28;8(5):e65179. doi: 10.1371/journal.pone.0065179 (PMC3665803; doi:10.1371/journal.pone.0065179)

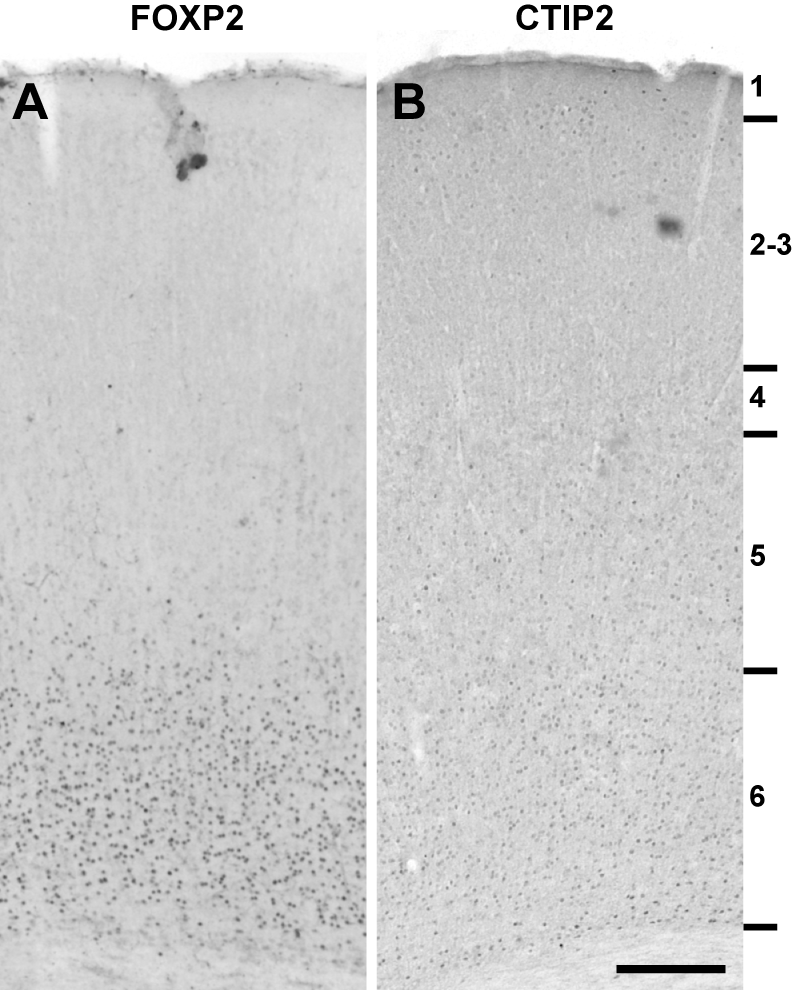

Supplement: Figure S1 — Laminar markers FOXP2 (A) and CTIP2 (B) in the somatosensory cortex. FOXP2+ and CTIP2+ neurons are found predominantly in layer 6, although CTIP2 inconsistently labels layer 5 and layer 2 neurons as well. Bar = 500 µm. (TIF) [file pone.0065179.s001.tif]
